# LandScan Global 30 Arcsecond Annual Global Gridded Population Datasets from 2000 to 2022

**DOI:** 10.1038/s41597-025-04817-z

**Published:** 2025-03-24

**Authors:** Viswadeep Lebakula, Kelly Sims, Andrew Reith, Amy Rose, Jake McKee, Phil Coleman, Jason Kaufman, Marie Urban, Chris Jochem, Carrie Whitlock, Mitchell Ogden, Joe Pyle, Darrell Roddy, Justin Epting, Eddie Bright

**Affiliations:** Oak Ridge National Laboratory, Oak Ridge, USA

**Keywords:** Geography, Scientific community, Research data

## Abstract

Oak Ridge National Laboratory (ORNL) annually develops the LandScan Global (LSG) dataset, a 30 arcsecond global gridded population dataset representing global ambient human population distribution. This multivariable dasymetric model disaggregates census counts within administrative boundaries using ancillary data. Each country’s distribution reflects cultural and socioeconomic patterns; manual validations yield a unique global dataset for assessing populations at risk. For over two decades, LSG has been a standard for estimating populations at risk, aiding U.S. federal government, academia and humanitarian organizations. During disasters such as the 2004 Indian Ocean tsunami and the 2010 Haiti earthquake and geopolitical crises such as the Syrian civil war and the 2022 Russian invasion of Ukraine, LSG supported scientific and operational communities in emergency response and recovery. In 2022, LSG datasets from 2000 onward were made publicly available through ORNL’s LandScan Portal. This data descriptor details our methodology and the application of geospatial science and machine learning to geographic and demographic data, highlighting uses in urban resiliency, emergency management, disaster response, and human health and security.

## Background & Summary

In the decades since the advent of digital mapping software in the 1990s, global gridded population datasets (GGPDs) achieved paramount importance with their ability to distribute populations to areas of expected human activity, which are not traditionally captured in census data. Gridded Population of the World (GPW), Global Rural Urban Mapping Project (GRUMP), Global Human Settlement Layer-Population (GHS-POP), WorldPop and LandScan Global (LSG) are regarded as the preeminent GGPDs by the scientific community^1–6^. These datasets all disaggregate census populations to a finer gridded resolution across the globe. However, they differ in modelling strategy, spatial resolution, frequency of dataset releases (only WorldPop and LSG provide annual estimates) and intended purpose^1–3,6,7^. As a result, the datasets may vary in their applicability to different scenarios because they were developed to address different problems^3,6,8^. Hence each of these datasets has advantages and disadvantages. This manuscript describes the methodology, illustrates the temporal uniqueness over time at a global scale, and informs users about the potential advantages and limitations of the LSG dataset.

GPW was initially developed by the National Center for Geographic Information Analysis (NCGIA) in 1995 at a resolution of 150 arcseconds and was the first effort to estimate global gridded population^1,9^. Since the release of the first version, GPW has been updated multiple times by the Center for International Earth Science Information Network and it is currently in the fourth version with an increased resolution of 30 arcseconds (approximately 1 km near the equator)^1,6,7,10–12^. This dataset can be augmented with geologic, economic and other social data, making it readily applicable to other disciplines^9,10^. The disaggregation of population across administrative units avoids using additional spatial datasets such as buildings or nighttime lights. Because these datasets are not inherently error free, excluding them minimizes the risk of introducing errors and uncertainties into the gridded population modelling process^3,6^. Furthermore, because the ancillary datasets are not used as an input to GPW, it is highly suitable for combining with other datasets. The key drawback of GPW lies in its areal weighting strategy which assumes an even distribution of population within administrative units^1,6^.

GRUMP^13,14^ and GHS-POP^4^ achieve higher spatial accuracy in population distribution with a similar binary dasymetric methodology by utilizing GPW and remotely sensed data^6,7^. The primary focus of GRUMP is to allocate the population among urban and rural areas at a 30 arcsecond resolution using nighttime lights^7,13,14^. However, the use of nighttime lights in GRUMP makes it vulnerable to blooming effects. Blooming refers to an overestimation of illumination from nighttime lights, where the brightness of nighttime lights spreads beyond their actual source^15,16^. Regions with lower nighttime lights and higher population allocation are especially difficult to distinguish from petroleum refineries, chemical plants and other sites associated with natural gas flares^6,17,18^. By contrast, GHS-POP disaggregates the population at 30 arcseconds and at a higher resolution of 3 arcseconds (approximately 90 m near the equator) based on built-up areas obtained from the Global Human Settlement Layer (GHSL)^4,6^. The shortcomings of this dataset are caused by incomplete settlement coverage data in GHSL. In the absence of building information, GRUMP follows a similar strategy to GPW by evenly spreading population across all cells. Moreover, errors in GPW may propagate to both datasets.

WorldPop uses random forest machine learning algorithms to estimate population density, followed by a dasymetric methodology to disaggregate the population, at 3 and 30 arcsecond resolutions^5,6,19,20^. In addition to gridded population estimates, WorldPop provides detailed demographic information such as population density, urban change, settlement growth, age and gender structures that are lacking in other datasets. Although annual estimates are available from 2000 to 2020, this disaggregation methodology was applied only for 2010, 2015 and 2020^19,21^. For the rest of the years, the population was interpolated by assuming a linear growth rate. This strategy may produce unrealistic results, particularly at the level of a grid cell.

A prototype of LSG was first developed in 1998 with the goal of producing a top-down estimate of populations at risk owing to natural and anthropological disasters^22^. This approach produced an ambient (24-hour average and unwarned) global population distribution dataset at a resolution of 30 arcseconds (roughly 1 km at the equator) by exploiting spatial data and imagery analysis technologies, in a multivariable dasymetric modelling effort to disaggregates census totals to population counts at a cell level^1,22,23^. This now annual release also distinguishes itself from other GGPDs by going beyond the traditional residential (nighttime) distributions and instead incorporates diurnal movements and travel habits into its modelling, capturing the unwarned nature of population distributions across a day’s 24-hour cycle^1,22,23^. Countries are modelled independently to produce significant variability throughout the model, ensuring unique customization that reflects the economic, physical and cultural factors exclusive to each region. Manual intervention in the form of edits is applied to account for internally displaced persons, rapid urbanization, unoccupied areas, war zones, out of date or inaccurate ancillary data, and transitory dynamics.

Since its inception, LSG has maintained its reputation as the U.S. federal government’s dataset of choice^24^ because of the extensive experience of Oak Ridge National Laboratory (ORNL) in developing population datasets, as well as access to regional and global scales of ancillary data and overhead imagery that no other producers had (especially in the earlier years) or might not yet have today. For over 50 years, ORNL has been involved in disaggregating census data using remote sensing, geographic information systems (GIS), dasymetric techniques and high-performance computing resources to address national security concerns. Additionally, ORNL’s relationship with government agencies like the National Geospatial-Intelligence Agency (NGA) (formerly the Defence Mapping Agency and the National Imagery and Mapping Agency), has facilitated access to otherwise restricted data and satellite imagery for processing and implementation into population models. For example, access to high-resolution commercial satellite imagery has allowed for the inclusion of data produced via advanced machine learning techniques such as computer vision and convolutional neural networks.

LSG is used by supranational organizations including the United Nations, World Health Organization, Food and Agricultural Organization, and federal, state, local and tribal agencies within the United States and governments of other countries^1,23^. Collectively, LSG has averaged approximately 2,400 downloads per month since April 2022 through the LandScan Portal (https://landscan.ornl.gov/). The users of these downloaded data include academics, researchers, government officials, policymakers, hobbyists, and others spanning the private, public, and nonprofit sectors. LSG has also been used in the areas of humanitarian response, climate change, counterterrorism, public health, policy planning, infrastructure allocation, homeland security, and disaster and risk analysis and to study coastal populations, identify shrinking cities and develop high resolution economic activity estimates^1,2,22–28^. Most notably, this dataset was put to practical use to identify and provide relief to potential victims during natural disasters such as earthquakes in Nepal and Iran and tsunamis in Sri Lanka and Indonesia. In 2006, the life-saving significance of the LSG dataset was recognized with an R&D 100 award^29,30^.

The success of LSG has led to the creation of two additional ORNL-produced gridded population datasets, collectively known as the LandScan datasets, which differ from LSG (and each other) in terms of the workflow and modelling strategy used to develop them as well as their spatial and temporal resolution^31,32^. LandScan HD^33–35^ and LandScan USA^36^ provide estimates at a much finer resolution (3 arcseconds) than LSG (30 arcseconds). Additionally, LSG is an ambient representation (average over 24 hours and unwarned), whereas LandScan USA is a daytime and nighttime distribution. LandScan HD also incorporates daytime and nighttime populations, but to produce the final product, these two populations are averaged to produce an ambient population. These datasets all differ in coverage availability; LandScan HD is only available for select countries, whereas LandScan USA includes all 50 states, Washington D.C., and all U.S. territories. Both LandScan HD and LandScan USA are the result of distinct, bottom-up approaches and act as stand-alone, proprietary datasets that are then incorporated into the LSG dataset when available. Further details about that process are explained in the next section.

As a proven community standard, LSG possesses several unique advantages for understanding at-risk populations compared with other global gridded datasets:

1. Ambient Population Modelling: Instead of estimating residential populations at the lowest available administrative boundary, LSG produces an ambient population for each cell, representing an unwarned and average distribution of population throughout the day. This approach addresses the question of where people might be at any given time over a 24-hour period in the event of a natural or human-caused disaster.
2. Integer Population Counts: Population counts within LSG grid cells are represented as nonnegative integers, rather than floating-point values. When the dataset was originally produced nearly 25 years ago, the decision to use whole numbers was primarily driven by storage limitations and computational constraints of that era. Despite advancements in computing technology, LSG reports population counts in integer format not only because this format is realistic for population distributions, but also because it will maintain consistency with the historic datasets.
3. Adaptable Methodology for Population Disaggregation: The population disaggregation methodology is applied annually, allowing for the incorporation of newly released ancillary data. This approach provides the agility to easily adapt to changes in the input datasets.
4. Country-Specific Models: LSG develops a unique model for each country, accounting for local factors such as habitation patterns and socioeconomic trends. This tailored approach ensures accuracy and relevance across diverse geographic contexts.
5. Manual Validation and Edits: Recognizing the intricate nature of human mobility and the potential variability in authoritative ancillary data, subject matter experts (SMEs) conduct manual validation and edits to refine distribution weights. This process involves analysing satellite and terrestrial imagery as well as leveraging place-specific data and knowledge to complement model output.

This data descriptor will offer valuable insights into methodologies that have evolved over the last two decades, and it will provide users with a necessary understanding to ensure appropriate implementation of each year’s LSG dataset. The following sections discuss in detail the measured approaches and strengths of LSG and cover some expected limitations. For example, while our consistent use of the dasymetric model remains a cornerstone, it is important to acknowledge the variations in methodology driven by evolving input data quality and scientific advancements make validation analysis and change detection difficult. A comprehensive examination of these methodologies and their implications are detailed in the next section.

## Methods

LSG’s disaggregation of census data into 30 arcsecond grid cells from 2000 to 2022 is the result of three individual modelling components:

1. Weighted coefficients serve as ratio multipliers generated from ancillary indicator datasets and applied to each cell.
2. Source zones are a combination of administrative unit population totals (as tabular data) and administrative unit boundaries (as spatial data).
3. Dasymetric modelling is the process of integrating the weighted coefficients and the source zones to produce a gridded population.

Since its initial release, LSG has used two distinct methodologies within these three components to adapt to the evolving landscape of geospatial data, particularly in terms of data precision and attribution. The original methodology, implemented from 2000 to 2014, used much coarser-resolution input data to compute the coefficients, which in turn results in undesired artifacts of population distribution (such as wider population distribution along roads than the road width). The original methodology also used more universal administrative and population data provided by a single source, influencing the source zones. However, around 2015, the growing availability and evolution of GIS data prompted a shift in methodology. Specifically, more spatially explicit data, such as building features, as well as attribute-rich data, such as points of interest, were used to improve coefficients weights. These changes reduced the commission errors that were so prevalent in the early years. Furthermore, the core processes for generating source zones and dasymetric modelling remained largely unchanged, but modifications were made to generate source zones more precisely. Specifically, source zones were initiated from country-produced internal administrative boundaries and population totals. Then, they were fitted to a single international boundary file and normalized to a unified global source of country population estimates.

Before each component is discussed further, it is important to emphasize that the overall methodology of LSG varies over time because it uses the highest quality input data available during each deliverable period. Although employing a constant methodology with the same set of ancillary inputs and updated census data every year is possible, this approach may detect neither the rapid nor the gradual changes over time caused by urbanization or destruction of existing settlement. Additionally, scientific and modelling improvements in input datasets over time result in a lack of consistent accuracy among the ancillary datasets, leading to inherent estimation errors in all GGPDs. Therefore, although LSG can perform change analysis as well as other GGPDs, users should avoid conducting change analysis at the cell level. Furthermore, the exact accuracy of LSG is difficult to report owing to the absence of ground truth datasets for all GGPDs at the grid cell level. Consequently, the ambient nature of LSG was validated using four different validation schemes, as discussed in Section 4, ‘LandScan Global Availability and Usage Notes’. For supplementary validation readings, use cases from the user community have analysed and reported the reliability of the LSG dataset^37,38^.

The following subsections discuss in greater detail the input datasets for the first two modelling components (coefficients and source zones), the equations describing the final component (dasymetric modelling) and the methodology shift, post 2015, that was caused by the evolution of GIS data.

### Target zones: coefficients

In the early years, LSG used a fixed set of ancillary datasets that covered every country throughout the world. These datasets comprised roads, slope, land cover, populated places, DMSP nighttime lights, exclusion areas, VMAP, and urban density factors^1,22,23^. This approach was limited by the coarser resolution of the datasets with complete coverage for the globe that were available 20 years ago compared with subglobal datasets. Furthermore, many of the specific sources used are unknown now and cannot be replicated. Although the historical inputs were an improvement for their time^22^, many of these sources are avoided today owing to inherent limitations and the existence of better data.

In the last decade however, a substantial increase in the precision, accuracy and diversity of input datasets was observed at individual subglobal levels (i.e. country-specific or region-specific datasets). Currently, these subglobal or local high-resolution datasets are used to improve the spatial fidelity of the population distribution by selecting the most recent and most precise data available for each geographic region. Datasets used to produce coefficients are provided in Table 1. The ‘Availability’ column refers to the spatial and temporal availability for each dataset to disclose which datasets have global, national or local coverage.

**Table 1:** Input datasets for use in coefficient modelling.

Dataset	Description	Availability	Resolution	Preprocessing	Source
Planet OpenStreetMap (OSM)	OSM is initiated to provide free and open-source GIS datasets. Building features from OSM are used in LSG.	Updated weekly for all countries.	Building polygons	Building centroids were summed by LSG cells	https://planet.openstreetmap.org/planet/
Multi-national Geospatial Co-production Program (MGCP)	MGCP produces topographic vector data such as boundaries, buildings, roads, utilities, and vegetation, etc. MGCP building data is used in LSG.	Available for over 30 countries	1° grid × 1° grid.	Building centroids were summed by LSG cells	Received directly from U.S. Department of Defence (not publicly available for download)
Microsoft building footprints	Microsoft applies computer vision technologies to Bing imagery to extract building footprints^44^.	Coverage available for most countries but omits large portions of many countries.	Building polygons	Footprints are converted into point file based on the centroid of the building.	https://www.microsoft.com/en-us/maps/building-footprints
Google open buildings	Building footprints are created by deploying a trained deep learning model on high-resolution satellite imagery^45^.	Available for sub-Saharan Africa and for select countries in South Asia and South-East Asia.	Building polygons	Footprints are converted into a point file based on the centroid of the building.	https://sites.research.google/open-buildings/
Global Human Settlement Layer	The Global Human Settlement Layer (GHSL) project produces GHS built-up surface dataset which is a raster that represents the spatial distribution of settlements.	Global coverage	100 m	Raster settlement layer to 3 arcsecond cells for refined coverage masking to lower coefficient weights in LSG cells that lacked settlement presence.	https://ghsl.jrc.ec.europa.eu/download.php
Oak Ridge National Laboratory’s building feature extraction	Building maps extracted from high-resolution satellite imagery using convolutional neural networks^39,40^	Developed for countries of interest that lack reliable and/or available building features.	Building polygons	Building areas are converted into a point file based on the centroid of the building.	Oak Ridge National Laboratory (Not publicly available)
Oak Ridge National Laboratory’s settlement maps	Binary spatial layer of human-built structures or settlement areas^41,42^.	Developed for early LandScan HD deliverables.	≤ 8 m	Raster settlement layer to 3 arcsecond cells for refined coverage masking to lower coefficient weights in LSG cells that lacked settlement presence	Oak Ridge National Laboratory (Not publicly available)
Oak Ridge National Laboratory’s PlanetSense points of interest	An open-source collection of Points of Interest data curated from multiple open sources^49^.	Global coverage except the U.S.	Points (latitude and longitude)	Filtered POIs related to building structures, buffered these POIs, and then rasterized them to fill in gaps in the GHSL data.	Oak Ridge National Laboratory (Not publicly available)
LandScan Global coefficients (previous year)	Global gridded likelihood weights by utilizing geographic datasets representative of human activity.	Global coverage	30 arcsecond × 30 arcsecond.	—	Can be obtained by using previous year’s LSG as described in Eq. 1
LandScan HD	Gridded ambient, daytime and residential populations for countries by utilizing high-resolution building features, occupancy, land use, and POI datasets. This model is independent of Census but normalizes bottom-up modelling to CIA WFB population.	Available annually for a few countries.	3 arcsecond × 3 arcsecond	3 arcsecond grid cells are aggregated to 30 arcsecond cells and used to identify distribution differences which later influence updates to the LSG coefficient	https://landscan.ornl.gov/
LandScan USA	Provides gridded daytime and nighttime populations for the USA by utilizing infrastructure and activity datasets. Census block level population is disaggregated using a hybrid approach.	Available from 2016 to 2021 for the USA.	3 arcsecond × 3 arcsecond	3 arcsecond daytime and nighttime population distributions are averaged then aggregated to 30 arcsecond cells and normalized to the State population totals in the CIA WFB.	https://landscan.ornl.gov/ (and HIFLD’s GeoPlatform)
LandScan Global (previous year)	Provides global gridded ambient population by utilizing demographic and geographic datasets. In an administrative unit, the census population is disaggregated using a top-down approach.	Available annually for all countries.	30 arcsecond × 30 arcsecond.	—	https://landscan.ornl.gov/

Each dataset above is ingested in parts of LSG every year, except for ORNL’s settlement maps (last used in 2017) and LandScan USA (2004–2012, 2016–2021). The settlement maps were replaced with more precise building feature type delineations (converted to centroids) and were available across more areas of the globe from multiple sources. The United States was prorated in the 2022 LSG dataset to reflect the updated population data but remained unchanged in the coefficients to avoid disrupting the high-fidelity distributions driven by the LandScan USA 2021 dataset.

Building features and settlement areas used in LSG include ORNL’s building feature extraction^39,40^, ORNL’s Settlement Mapper output^41,42^, Open Street Map (OSM)^43^, Microsoft’s building footprints^44^, Google’s building footprints^45,46^, Multinational Geospatial Co-production (MGCP)^47^, GHSL^48^, and PlanetSense points of interest^49^. OSM and MGCP data are available to download in point files without the need for further processing. However, accurate building information is not available in these datasets for all countries. For countries with inaccurate or unavailable open-source data, building features are extracted from satellite imagery using the computer vision technique (U-Net) developed at ORNL. Later these footprints are converted into points representing the centroid of the buildings to update the raster coefficient model. Because the previous year’s LSG is one of the inputs to the current LSG, residuals from the initial version of LandScan that used input datasets such as roads, slope, land cover, populated places, nighttime lights, exclusion areas, and urban density factors are potentially still present in the current version in data-poor areas such as Gran Chaco, Bolivia.

### Source zones

#### Demographic/Enumeration data

Demographic data include population totals for national and/or subnational administrative units. Every year, new population data are collected, if available, for each country at an appropriate administrative unit that allows for an ambient distribution (explained further in Section 2.2.2,’Geographic Boundary Data’), either from the country’s own census/microcensus or surveys conducted by independent organizations such as nonprofits or research organizations. To create a consistent global baseline population, all subnational populations are then normalized at the country level using midyear population estimates obtained from the C.I.A. World Factbook (CIAWFB)^50^. Table 2 lists these inputs in the same format as Table 1.

**Table 2:** Input datasets for use in tabular source zone modelling.

Dataset	Description	Availability	Resolution	Preprocessing	Source
Subnational Population	The population of administrative units.	Not available annually. Varies for countries depending on Census.	Varies from the block level to the county level.	The subnational population is normalized by CIA World Factbook country population counts.	Varies, but typically websites for national statistical agencies
C.I.A. World Factbook (CIAWFB)	National population estimates are provided by The United States Census Bureau’s International Database and are based on censuses, registration systems, or sample surveys pertaining to past and future trends.	Available annually for over 95% of countries.	Country level.	Subnational census totals are normalized so that the country’s population total matches the CIAWFB population.	https://www.cia.gov/the-world-factbook/about/archives/

#### Geographic boundary data

Datasets that fall under the geographic source zones are administrative boundaries that can come from many diverse sources. These boundaries maintain the disaggregation of populations within an area and do not necessarily represent the finest delineation available. For example, if the United States enumeration units are considered as small as a census tract, block or block group, then populations begin to be confined in residential-only areas, ignoring the ambient nature of population dynamics throughout the daytime. Therefore, boundaries are selected towards larger boundaries like villages or city levels to properly reflect mobility ranges throughout a given 24-hour period. Although the focus on finding country-driven interpretations of boundaries is crucial to ensuring that these boundaries agree with their own reported population numbers, these geographic source zones are used only for internal boundaries. Each country is then fitted to a single global source of international lines from the Large Scale International Boundaries dataset^51^. This multilayered process allows for population data to remain true to the designated geographic enumeration unit while removing disputed interpretations of international boundary ownership. Table 3 lists these inputs in the same format as Tables 1 and 2.

**Table 3:** Input datasets for use in geographic source zone modelling.

Dataset	Description	Availability	Resolution	Preprocessing	Source
Boundaries of administrative units	For each country, internal boundaries are sourced from the official country data. In the absence of country specific source, we used available Global Administrative Unit Layers (GAUL) that are legacy boundaries from LSG early years.	We perform annual checks on all countries for new boundary changes. Existing data is updated to reflect the new boundary changes.	Administrative unit.	Gridded to 30 arcsecond cells.	Individual country websites or https://data.apps.fao.org/map/catalog/srv/eng/catalog.search?id=12691#/metadata/9c35ba10-5649-41c8-bdfc-eb78e9e65654
Large Scale International Boundaries	Office of the Geographer and Global issues at the U.S. Department of State provides international boundary lines.	Available for all countries with varying update frequency.	Country boundaries (minus coastal lines)	Gridded to 30 arcsecond cells.	https://geonode.state.gov/layers/catalog:geonode:LSIB

### Dasymetric modelling

The final component is the dasymetric model, which relies on source zones and target cells to produce population distributions across a 30 arcsecond raster. The source zones contain demographic tabular data (populations) and geographic data (boundaries and administrative units). Target cells are coefficient weights (built by years of ancillary dataset inputs) that distribute shares of population counts. What makes LSG unique is that each country has its own model, which has been developed independently over the past 20 years^1^. Consequently, replicating the exact components is impossible. However, coefficient weights can be simulated by incorporating various source zones with the LSG datasets. Coefficient weights for all the grid cells of an administrative unit can be calculated as shown in Eq. (1).

1 $${{Pre}{v}_{c}}_{{ij}}=\frac{{{Pre}{v}_{p}}_{{ij}}}{{Pre}{v}_{{AdminPop}}},$$

where ${{Pre}{v}_{p}}_{{ij}}$ is the previous year LSG population of a grid cell in the *i*th row and *j*th column, and ${Pre}{v}_{{AdminPop}}$ is the previous year population total of the administrative unit. Although reverse engineering to replicate coefficients may not result in exact weight values per cell, Eq. (1) represents the relative magnitudes among the cells. Dasymetric modelling can be used with these reverse-engineered coefficients and the CIAWFB country population estimates to obtain the same LSG population distribution.

Apart from the above-mentioned dataset categories, the previous year’s gridded source zones are used to develop new source zones for the current year. The source zone consists of both demographic (the previous year’s population) and geographic (boundary) information at the subnational level for all the countries. To achieve the ambient nature of LSG, buildings are not differentiated based on utility (such as residential and commercial). Furthermore, use of the finest census population counts and boundaries is avoided to minimize constraint to a very small area and, in most cases, to yield a distribution of residential population.

### Methodology to estimate global gridded population from 2000 to 2014

**Fig. 1:**
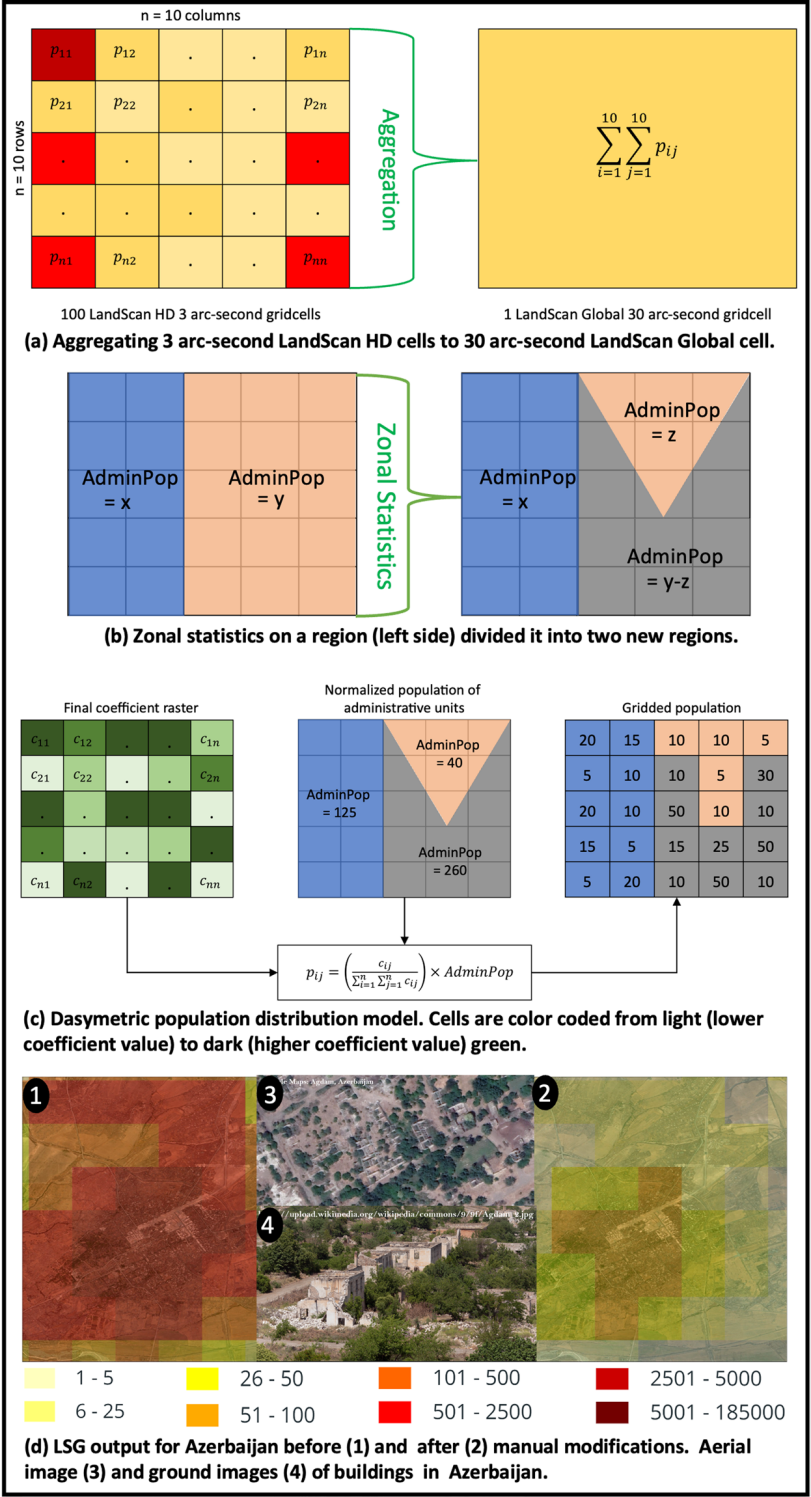
Subprocesses in LandScan global gridded population estimates. The terms p_ij_ and c_ij_ represents population and coefficient of a cell located at the ith row and jth column in the 2D grid. For visual clarity, this concept is illustrated using a simple 5 × 5 grid raster.

A single global model to accurately estimate the gridded ambient population of the world that can account for disparate data availability and place-based differences such as cultural settlement practices of all countries is not feasible^1^. Hence, the population for each country is estimated separately, and they are later combined to produce LSG (refer to Algorithm 1 in supplements). Leveraging high-resolution (3 arcsecond) gridded population estimates for the group of countries using LandScan HD or LandScan USA, the LSG methodology changes for each country based on their availability. Unlike LandScan HD (ambient population), LandScan USA provides daytime and nighttime population counts separately. So, for the United States, the 3 arcsecond nighttime and daytime population rasters are aggregated to 30 arcsecond cells (Fig. 1a), and the arithmetic mean of these rasters is calculated to obtain the ambient population from LandScan USA.

**Fig. 2:**
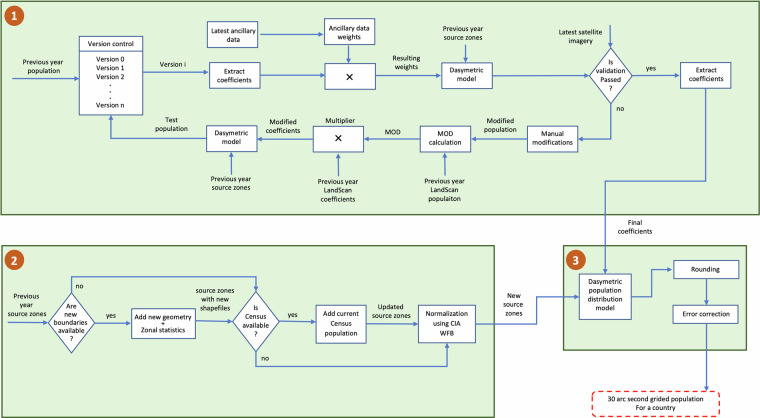
Architecture to estimate the gridded population estimation procedure for countries without LandScan HD or LandScan USA from 2000 to 2014.

The procedure to estimate the population for the remaining countries without LandScan HD or LandScan USA involves a three-part process, and each part represents a distinct workflow (Fig. 2). The first part starts by labelling the previous year’s LandScan population (*PrevPop*) for a country as *Version* 0. The latest available ancillary datasets are applied to *Version* 0 to recalculate the underlying weights by converting new ancillary datasets to their respective weight rasters at 30 arcsecond resolution. These ancillary weight rasters are multiplied with the original coefficient (weight) raster of *Version* 0. The resulting weight raster, along with the source zone population, was used to disaggregate population based on the dasymetric model, yielding an updated *Version* 0. To validate how the new input data influence the previous distribution (*PrevPop*), updated *Version* 0 is visually inspected by overlaying it on high-resolution satellite imagery to evaluate its usefulness. A version is considered invalid if imagery analysts detect inconsistencies during these inspections. For instance, in a grid cell, inconsistencies arise when population is missing despite visible buildings in the imagery associated with that particular grid cell. These inconsistencies are often caused by latencies in converting newly captured satellite imagery into ancillary data, whether through manual building feature extraction or automated methods using computer vision algorithms. *Version* 0 frequently fails this evaluation because the ancillary data may not reflect recent changes in the built environment. Additionally, the ancillary datasets that are used for population distribution can contain both spatial and temporal inaccuracies^1,3,6,8^, that can vary across geographies and time periods. The accuracy of ancillary data obtained from remotely sensed sources depends on the quality of sensors and the accuracy of algorithms that were used for data extraction (e.g. building footprints are extracted from satellite imagery)^8^. This research we used building footprints from different organizations, including Microsoft, Google, and ORNL. The primary reasons for incorporating different building footprints are the varying accuracies of these footprints across the regions and gaps in building footprints caused by incomplete extraction^52^. Microsoft and Google perform semantic segmentation using computer vision algorithms to classify pixels in satellite imagery to either building or non-building associated pixels^52^. This classified pixel information was later used to generate building footprints. Both Microsoft and Google reported an approximate recall of 70%. In countries where Google or Microsoft building footprints are either unavailable or inaccurate, ORNL generates footprints using high-resolution satellite imagery processed through similar computer vision algorithms^40,41^. In Taizz, Yemen, ORNL building footprint algorithms achieved recall of over 71%. Therefore, visual validations or manual inspections are necessary to identify inconsistencies when detected manual modifications (discussed with examples in Section 3.2, ‘Manual Validations and Modifications’) can be used to correct them, in turn increasing both spatial accuracy and population distributions^1^. Additional investigations are made using the latest satellite imagery or knowledge about recent global events. Finally, a SME establishes manual modifications, as a multiplier, on *Version* 0 to either reduce or increase the populations, resulting in a modified population (*ModPop*). These modifications can be unique to an area in question and/or to individual cells. The purpose is to supplement quantitative data with qualitative adjustments to better represent the magnitudes between cells, capture unrepresented distributions, or correct erroneous ancillary data areas. In a modified population raster, for each cell *i, MOD* (Eq. [2]) is calculated and is later multiplied by the previous year’s LandScan coefficients to generate modified coefficients.

2 $${{MOD}}_{i}=\frac{{{ModPop}}_{i}}{{{PrevPop}}_{i}}.$$

Modified coefficients obtained from this subprocess for a country are identified and updated (*CoeffUpdated*) for all *a* administrative units (each with a 2D grid of *r* rows and *c* columns). Each administrative unit’s updated coefficients, along with the source zones containing boundary and population for the previous year (${{AdminPop}}_{{prev}}$), are used by the dasymetric population model (Eq. (3) and Fig. 1c) to generate a test population (${{TestPop}}_{{country}}$) at 30 arcsecond resolution for the country. The result is stored as *Version* 1.

3 $${{TestPop}}_{{country}}=\mathop{\sum }\limits_{j=1}^{a}\left(\frac{{{CoeffUpdated}}_{{rc}}}{\mathop{\sum }\limits_{r=1}^{n}{\sum }_{c=1}^{n}{{CoeffUpdated}}_{{rc}}}\right)\times {{AdminPop}}_{{prev}}.$$

*Version* 1 may not pass this step, especially in regions where local knowledge is vital, such as the presence of ghost cities, water bodies and national parks. For example, Aghdam, Azerbaijan, was found to be a ghost city with vacant buildings when manual validations were performed using high-resolution imagery. To properly reflect the lack of people living in this war-torn region, a very low modification multiplier was used to reduce the population (Fig. 1d). Conversely, populations may be increased in regions when residential population (census) is restricted within smaller administrative units despite known higher human activity during the day, as is the case with downtown Washington D.C. The remaining steps are carried out to produce a new test population, *Version* 2. For a country, several versions may be computed before it passes the validation step. The corresponding coefficients of a version that passes the validation are considered as final coefficients. Relative accuracy of the population distribution can be increased through these manual adjustments by either allocating people where they should be or by removing people from where they should not be. LSG incorporates both aspects to increase accuracy.

The second part of the process (Fig. 2) builds on the source zones of the previous year to output new source zones with updated boundaries and population counts for all administrative units in a country. Subnational geographies can change, including when multiple regions or sub-regions are divided and/or merged or where boundaries have been redrawn altogether. To update the boundaries of these geographies, old geometries (region[s] before change) are replaced with new geometries (region[s] after change) in the source zones. Zonal statistics are then calculated using the LandScan population from the previous year to obtain a baseline total population for the new region(s) (Fig. 1b). If a country produces a census for the current year, then it updates the source zones population at the administrative level. Otherwise, the previous source zones population is used. As a next step, the updated source zones population at the administrative level (*AdminPopUpdated*) is normalized using Eq. (4) and the country population estimate of the current deliverable year, which is provided by the CIAWFB (${{CI}{A}_{{WF}{B}_{{Pop}}}}_{{current}}$). The result is a normalized population (${{NormPop}}_{{admin}}$) for all *a* administrative units in a country. In addition to adjusting for intercensal population growth and decline, this step helps to bring all countries to a common scale because different countries use different methods to estimate the population.

4 $${{NormPop}}_{{admin}}=\left(\frac{{{AdminPopUpdated}}_{i}}{\mathop{\sum }\limits_{k=1}^{a}{{AdminPopUpdated}}_{i}}\right)\times {{CI}{A}_{{WF}{B}_{{Pop}}}}_{{current}}.$$

Because the first and second parts of the process depicted in Fig. 2 are independent of each other, for time efficiency, they are executed in parallel. Finally, the outputs of the first part (final coefficients) and second part (new source zones) of the process are provided as input to the third part of the process. The population of each administrative unit is distributed across all the grid cells of that given administrative unit (*r* rows and *c* columns) based on coefficients (*Coeff*) using the multivariable dasymetric model (Eq. [5] and Fig. 2c). This model distributes more people to cells with a higher coefficient value.

5 $${{Pop}}_{{rc}}=\left(\frac{{{Coeff}}_{{rc}}}{\mathop{\sum }\limits_{r=1}^{n}{\sum }_{c=1}^{n}{{Coeff}}_{{rc}}}\right)\times {{NormPop}}_{{admin}}.$$

The dasymetric model results in decimal values because fractional terms are present. These terms are converted into integers by performing rounding and error correction. Rounding involves approximating the decimal values to the nearest integer using the half round up method. This process may result in estimate errors, so an error check is performed by summing the population of all the cells in the country model and comparing it with the reported country total population from the CIAWFB. If they are not equal, then the error is calculated by taking the difference between them. This discrepancy is then subtracted or added to the total population of the administrative unit with the largest population. This correction mitigates the ratio effect between each source zone and the underlying census numbers that drive these normalized totals. Countries with a significant number of administrative boundaries often exhibit more pronounced rounding errors, sometimes reaching upwards of 100 people. In such instances, the discrepancy is distributed across multiple units with the largest populations, thereby alleviating the effect on a single source zone. However, error differences are typically less than 10 people and have very little influence when corrected with the single, largest populated unit.

LandScan HD may result in gaps when building data are missing because distribution occurs at the building level. To address these gaps, an LSG output, for the same country, is generated for comparison using the methodology provided in Fig. 2. The output is stored as raster *m* (refer to Algorithm 1 in supplements). Raster *l* was computed by aggregating 30 arcsecond cells from LandScan HD (Fig. 1a), which is later compared with raster m by calculating percent change. The cells with higher percent change may represent an actual change or a gap in the LandScan HD population data. Manual validations are performed in these cells using the latest available satellite imagery. If these validations identify the gaps in a region, then cells in raster l are replaced with cells in raster *m*. The resulting replaced raster is the LSG for the country. Some of the LSG country level boundaries are not spatially aligned with either LandScan USA or LandScan HD because they have different resolutions (3 arcsecond vs. 30 arcsecond),yielding unaccounted populations. In such scenarios, the population of unaccounted LandScan HD and LandScan USA cells is assigned to the nearest LSG administrative unit before normalization is performed.

### Methodology to Estimate Global Gridded Population from 2015 to 2022

**Fig. 3:**
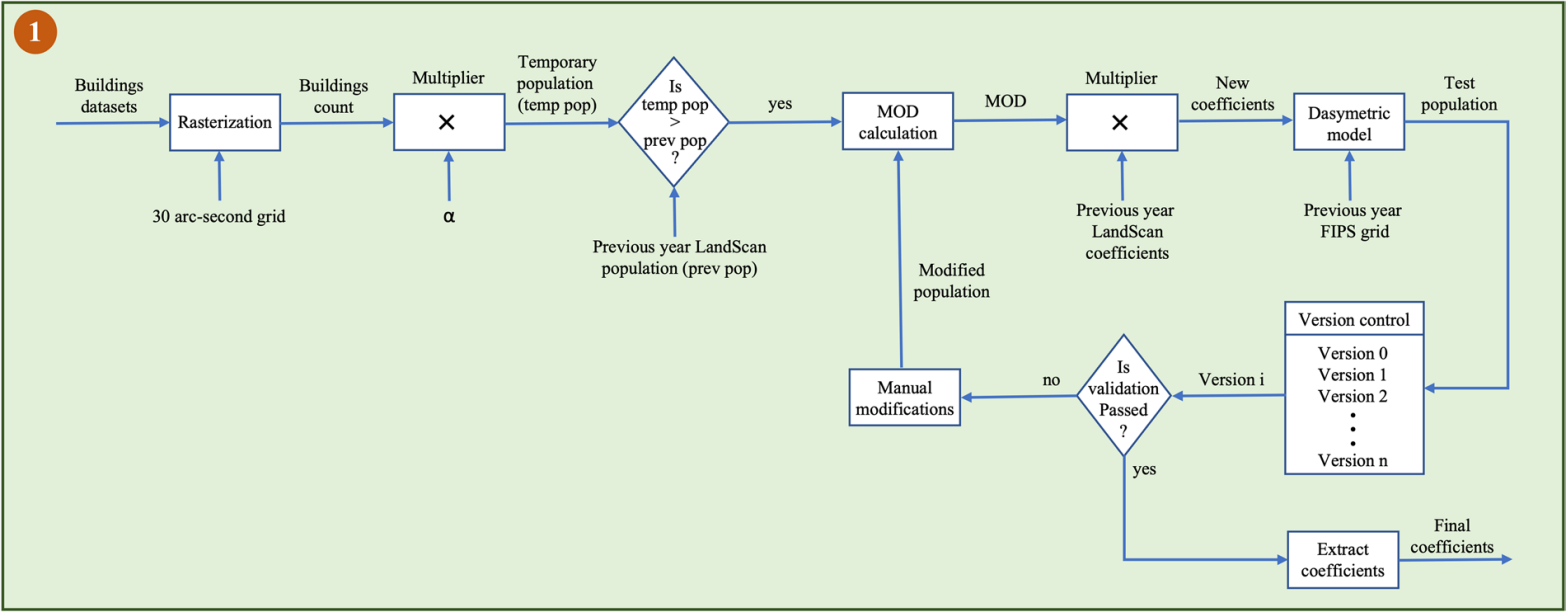
Architecture to illustrate the gridded population estimation procedure for countries without LandScan HD or LandScan USA from 2015 to 2022.

With the introduction of building footprints in 2015, the LSG methodology remains the same for countries with the availability of LandScan HD or LandScan USA datasets. For other countries, changes were made to the first part of the process (Fig. 2) to generate final coefficients. A graphical representation of this change is provided in Fig. 3. In the first step, building footprints in point format are converted into a raster using a 30 arc-second grid of a country. Each of the grid cells in the raster was then assigned a value equal to the number of buildings within that cell. A temporary population (TempPop) is obtained for all the grid cells by assigning a constant (*α*) number of people per building. This constant changes for each country. For LandScan HD countries that were ingested into LSG, *α* is obtained from the Population Density Tables^53–55^, whereas for other countries *α* is from the previous knowledge of that specific region. The current year’s temporary population is compared with the previous year’s (*PrevPop*) LSG population for all the grid cells. In a grid cell (*i*), if the temporary population is less than or equal to the previous population, then the LSG coefficient of the previous year is assigned as the updated coefficient to the cell *i*. Otherwise, *mod* is calculated using Eq. (6) and is multiplied by the previous year’s LandScan coefficient to generate a new or updated coefficient for the cell *i*.

6 $${mo}{{d}}_{i}=\frac{TempPo{p}_{i}}{{Prev}Po{p}_{i}}.$$

A dasymetric model (Eq. [2] and Fig. 1c) was implemented at the administrative level with the updated coefficients (*CoeffUpdated*) and previous year source zones to generate a test population (${{TestPop}}_{{country}}$), stored as *Version* 0. The previous year’s source zones are used to avoid the expected fluctuations driven by new demographic data and to understand exactly how the updated coefficients changed. For example, if population of cells increased excessively when the previous year source zones stayed the same, then one of two things occurred:

1. The previous distributions were wrong, and the new ancillary data corrected the cell estimates, or
2. The new ancillary data were inaccurate, and the previous distributions were more appropriate.

If adjustments are necessary to the above scenarios, or, if during the visual inspections other areas are identified with commission/omission errors, then a supplementary layer is created to capture manual modifications. This layer, denoted as MOD in Eq. (2), serves as a multiplier to the updated coefficients (*CoeffUpdated*). This multiplier adjusts cell weights higher (>1.0) or lower (<1.0), influencing the next step to generate a new population (*Version* 1). This loop continues until a version successfully passes validation, as determined by an SME, and the remaining coefficient becomes the final coefficient.

This annual and supplementary process to involve humans in the loop remains one of the greatest limitations to replicating LSG, as well as explaining the historical improvements and amendments. However, these modifications also contribute to LSG’s advantages over other GGPDs: the model has compounding SME interventions that further validate and rectify evolving or deficient geospatial inputs. In essence, each iteration of the LSG datasets is perfected year after year, enhancing the likelihood that distributions have been examined in many areas throughout the world.

## Data Records

The LandScan datasets from 2000 to 2022 are freely available at figshare 10.6084/m9.figshare.28439699^56^. LSG datasets are in the WGS84 datum geographical coordinate system with a raster grid of 933,120,000 (21,600 rows and 43,200 columns) 30 arcsecond cells spanning 90° north to 90° south and 180° west to 180° east. Each dataset contains an attribute table with ‘Value’ and ‘Count’ columns. The Value field represents the population count in a cell, and the Count field specifies the number of cells with the same population value. Multiplication of these two fields can allow for a total population when summed. In addition to the repository, our datasets are also available for users to download and visualise at https://landscan.ornl.gov/.

## Technical Validation

GGPDs are difficult to validate and impossible to verify owing to the absence of ground truth on a global scale at the grid cell level. Currently, no single strategy is appropriate for validating all GGPDs across the globe because the GGPDs are developed with different objectives. The validation complexity further increases for LSG because no ambient population such as census tract (residential population) is available at a coarser level than grid cell^1^. The primary objectives of LSG are to develop ambient populations and to improve in population distribution modelling over time through new census/surveys and geospatial ancillary data. To this end, the presence of equal population distributions (referred to as ‘peanut butter smears’), SME updates (manual edits), improvements over time and validating ambient representation are evaluated to track the progress of LSG in achieving these objectives.

### Peanut butter smears

**Fig. 4:**
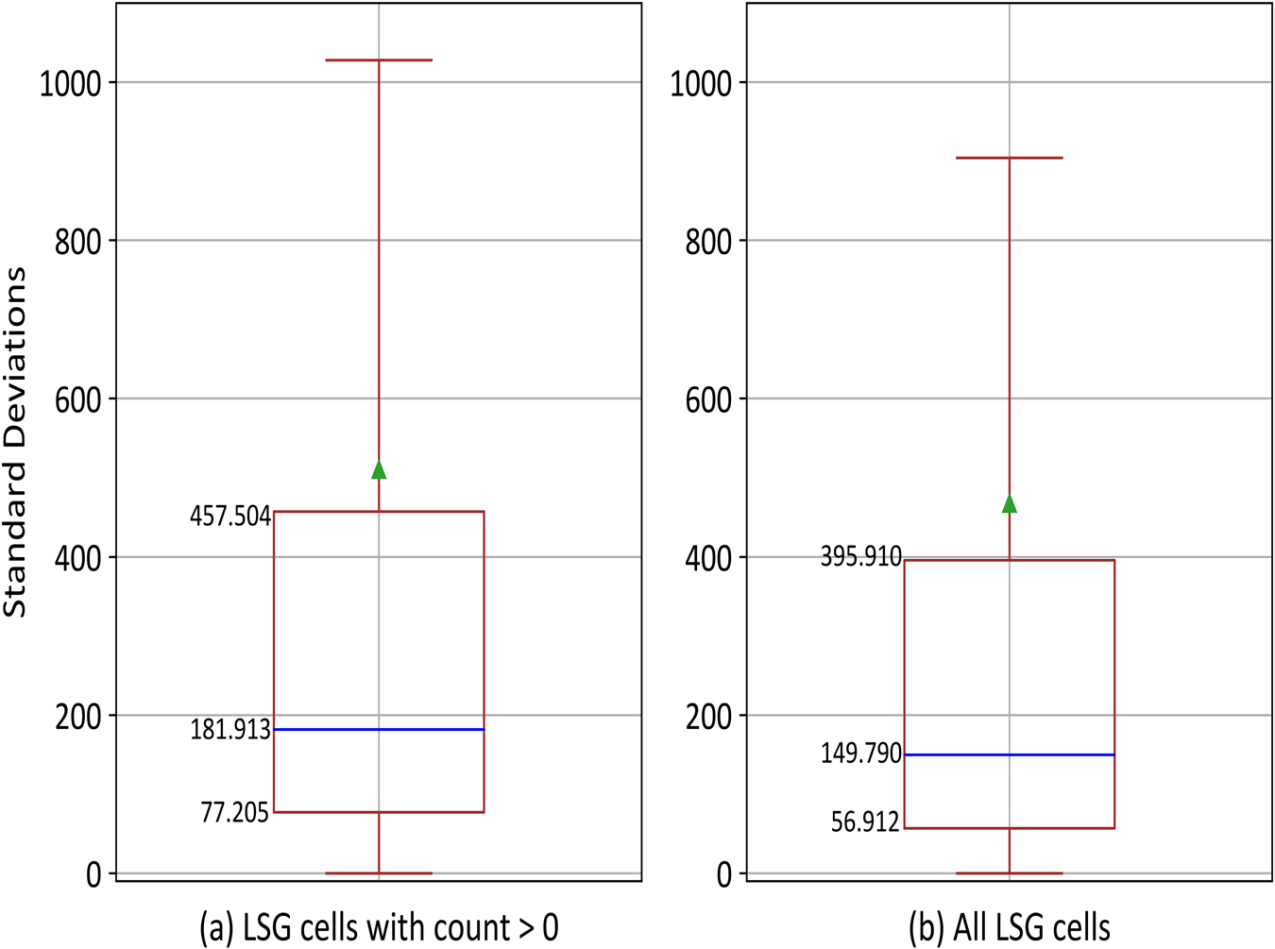
Box plot (without outliers) of standard deviations of population counts for all administrative units across the globe for the LSG 2022 dataset. For each administrative unit, standard deviation was calculated by considering (**a**) cells with population count greater than zero and (**b**) all the cells. Median and mean standard deviations are represented as blue horizontal line and green triangle, respectively.

One of the features of LSG high-resolution population distributions is that the cells in the administrative units do not exhibit peanut butter smears (the same number of people across LSG cells within an administrative unit). Standard deviation (SD) is used to quantify peanut butter smears because this value can explain variance across the cells within each administrative unit. Put differently, if an administrative unit has the same population count for all the cells, then it’s SD is zero, indicating the presence of a peanut butter smear across it. Higher values of SD represent larger differences in population counts among cells. The most recent LSG deliverable is 2022 with 76,513 administrative units (Fig. 4). To investigate the skewness caused by cells with zero count, the SD is calculated by dropping cells with zero population (Fig. 4a) and including cells with zero population (Fig. 4b). A significant number have a SD greater than zero and only 16 (≈0.02%) administrative units have a zero SD; the distribution of SD for the administrative units for the globe is skewed upwards with a mean of 510.05 (Fig. 4a). Over 75% of administrative units have SD greater than 77.205, over 50% have SD greater than 181.913 and over 25% have SD greater than 457.504. Similar distribution of SD is observed among the administrative units when the cells with zero count are included but with relatively smaller interquartile range, varying from 56.912 to 395.910 (Fig. 4b).

### Manual validations and modifications

One major effort is the incorporation of subject matter expertise into population modelling beyond ancillary input data. Instead of assuming the output is complete after the dasymetric modelling process, manual validations use satellite imagery and human activity knowledge about an area to make appropriate adjustments to the population estimates. As population features are extracted from satellite imagery into ancillary data (e.g. building footprints), the LSG uses ancillary data to disaggregate administrative unit population. However, generating ancillary data for the whole globe requires significant time and compute resources. Consequently, ancillary data are likely incomplete for any given year owing to the latency involved in ancillary data extraction from source satellite imagery. Put simply, even though satellite imagery is available for the current year, ancillary data are available for the previous year. Moreover, ancillary data are not error free, as discussed in previous sections. Therefore, manual validations and modifications can aid significantly in scenarios such as rapid response and areas with incorrect or incomplete data inputs. Some examples for cases that fails manual validations and require manual modifications are discussed in this section.

First, the Aghdam region of Azerbaijan contained a ghost city with vacant buildings. This city was not recorded in the ancillary data. When superimposed with the latest available high-resolution satellite imagery to perform manual validations, this region failed the validation. So, a very low modification multiplier was used to reflect the actual population distribution in this region. The population distribution rasters with and without manual modifications are presented in Fig. 1d.

Second, owing to the time delays of remotely sensed data, urban growth in areas such as Bahrain and (Cairo) Egypt were not captured frequently enough to reflect recent population changes in the model. Therefore, as described in Section 2, ‘Methods’, a supplementary layer is created by the SME as a multiplier to the coefficient raster for the purpose of increasing (>1.0) or decreasing (<1.0) the necessary cell weights. Once a test population output is approved by the editing SME, the model is theoretically more representative and circumvents these limitations.

Third, in Africa, in the first version, the population distribution was allocated to uninhabitable grid cells because the building footprint extraction was not 100% accurate^57^. For example, during manual validations, a rock in the desert was found to be mislabelled as a building. The dasymetric model assigned the population for that grid cell by considering the mislabelled building, which was manually modified, and the population of the grid cell was set to zero.

Another example for which manual modifications often occur is during wars or natural ddisasters. The Russian invasion of Ukraine is the latest precedent for which the model must reflect the movements of internally displaced persons and refugees leaving the country. When population movement is not captured in latest available census, manual modifications are applied to source zone population as well based on media reports. The latest satellite imagery and documented damaged areas are also used to alter the estimates.

Although manual validations may be labour intensive, we the advantages of incorporating humans in the loop outweigh the sole use of globally scaled modelling techniques that utilize the latest spatial data. In fact, LSG SMEs emphasize the importance of eyes on the data at various processing stages by actively researching and scrutinizing newly produced and openly distributed spatial data each year. For instance, a portion of evaluated data may not be utilized (e.g. too small of administrative boundaries or out of date land use data) or may be selectively used for specific regions or purposes (e.g. refugee camps). Furthermore, potential new input datasets often provide value only through additional content or spatial editing, such as adjusting datasets to get them in the right location or making corrections to an attribute field in order to capture internally displaced persons. This meticulous process guarantees that new data are never integrated indiscriminately without consideration of inherent errors before integration. Furthermore, it necessitates an annual review to each of the modelling components to properly depict the dynamic nature of the world. This substantial effort invested in more than 20 years of modifications is a key distinguishing characteristic that separates LSG from other GGPDs and is one of the fundamental aspects to its methodology.

### Validation of improvement over time

**Fig. 5:**
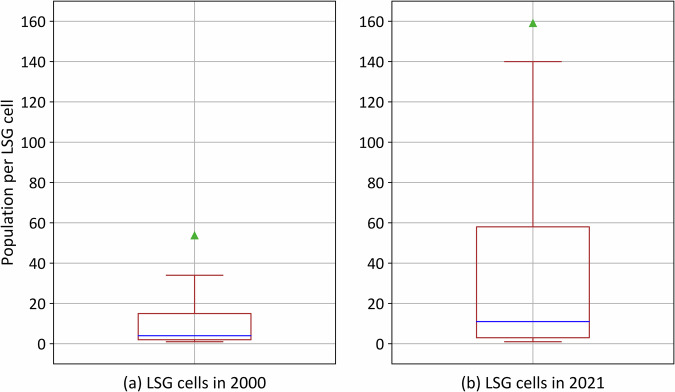
Box plot (without outliers) of cell population counts for all the LSG cells in (**a**) 2000 and (**b**) 2022. Median and mean population counts are represented as blue horizontal line and green triangle, respectively.

The range of per-cell population significantly increased from 2000 to 2022 (Fig. 5) along with an addition of over 9.3 million active LSG cells (Table 4). However, a substantial drop (decreased by 58.053%) occurred in cells with population count greater than zero (Table 4). Therefore, the ability to identify and remove populations in regions that potentially are not occupied by people improved over time. In other words, this increase in the presence of zero-population cells implies a reduction in the peanut butter smears (or commission), contributing to more precise distributions.

**Table 4:** Number of cells with different population counts for years 2000 and 2022.

Year	Population per cell	Percentage	Number of cells
2000	Zero	63.324%	195,676,647
2000	Greater than zero	36.676%	113,332,454
2000	All values (≤0)	100%	309,009,101
2022	Zero	85.068%	270,834,173
2022	Greater than zero	14.932%	47,539,247
2022	All values (≤0)	100%	318,373,420

### Ambient validations with the U.S. census

In the United States, the dasymetric model disaggregated the population of counties to the grid cell level. Even though comparing the residential (census) population with the ambient (LSG) population cannot directly validate the accuracy of LSG, it can provide insights into the ambient nature of LSG. To compare these datasets, the population was aggregated within a census tract, which is finer resolution than the U.S. counties. If LSG only contains residential population, then fewer disparities should exist between the datasets with higher correlation. In other words, if the summed LSG cells in a census tract are less than the corresponding census tract sum, then people are properly removed from their residential homes to daytime locations (e.g. schools, factories, commercial) of all the LSG cells that fall under each of the U.S. census tracts^57^ for the year 2021. This information is are not typically captured in traditional census data. Among the set of *c* census tracts (${t}_{1},{t}_{2},{t}_{3},\,\ldots \ldots ..{t}_{c}$), a considerable disparity is observed between datasets with a mean absolute deviation (Eq. [7]) of 1625.677.

7 $${Mean\; Absolute\; Deviation}=\frac{{\sum }_{t=1}^{c}|{{Census\; population}}_{t}\,-\,{{LSG\; population}}_{t}|}{c}.$$

**Fig. 6:**
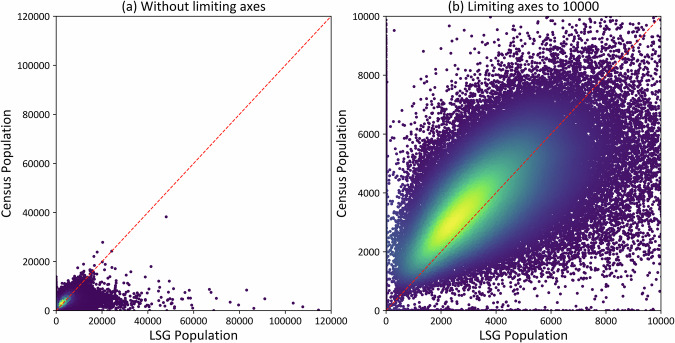
Scatter plot of LSG and Census populations of 2021 at tract level in the United States (**a**) without limiting the axes and (**b**) with limiting axes to 10,000. Points in the plots are coloured from yellow (high density of data points) to purple (low density of data points). The red dashed line is a 45° line.

Scatter plots are provided with and without limiting axes to examine the nature (over or underestimates) of disparities (Fig. 6). For some tracts, LSG overestimated by large margins. This result is expected because these metropolitan areas contain more economic and business activities and lower residential population (Fig. 6a). This type of population dynamics is captured in ambient estimates but not in residential population. From Fig. 6b, a large frequency of points (in yellow) shows how LSG is underestimating in census tracts. This result supports ambient distributions by moving people away from residential-only areas. However, some data points indicate a strong agreement between census and LSG estimates because the residential population is a part of the ambient population; these areas are often multiuse spaces (i.e. apartments or residential buildings on upper floors with commercial offices on the first couple floors).

## Usage Notes

The primary function of gridded population datasets is to allow users to extract the population of a region anywhere within or even crossing administrative units. For quick assessments, the LSG website is interactive and allows users to draw a polygon anywhere around the globe to gather population density (per square kilometre) and counts within that polygon. For further geospatial analysis, the downloaded TIFF file can be compared with other GIS data or converted to vector formats for regions of interest. Because the raster is initially in a geographic coordinate system, the data are not projected. Therefore, they represent population counts rather than density. In fact, the cell can be measured at 1 km at the equator but does not retain the same shape from the equator to the North or South Pole. Each cell becomes smaller the closer it is to the poles because of the arcsecond measurements.

To find the population density, users must perform a series of recommended steps to avoid errors such as on-the-fly reprojecting and cell creeping.

1. Convert the raster to a point file based on the Value (population) field.
2. Project the points to the desired projection.
3. Rasterize the points to a factor of the desired output cell size using a unique ID for each point. This output will produce a raster with an incrementing value of cells.
4. Join the point gridcode (population) field back to the new raster’s value field (unique ID).
5. Aggregate the compressed raster (using a multiplier of the third step) to the final output size.

LandScan Global’s primary use is unique from other GGPDs because it reports an ambient population count to estimate at-risk or unwarned populations (motivated by understanding the presence of populations throughout a 24-hour period or not captured in traditional census data). This dataset is not suggestable for analysis that explicitly focuses on residential-based locations because the model incorporates places of all activities, such as where people go to work, school, shop, eat, and recreate. In fact, LSG covers any potential space with human presence (including open spaces) to support emergency response in times of disasters, which can occur at any moment of the day. By averaging the distributions of populations over a 24-hour period, a more realistic coverage of full potential activity spaces is captured. A single temporal snapshot of where people reside at night or where they visit during the day will not fully support disaster relief efforts, especially if the disaster occurs during the daytime. Most other GGPDs capture residential populations accounting for nighttime (or populations at home in bed). The ambient or unwarned population estimates provided by LSG can be used in wide range of studies such as economic activity, national security, counterterrorism, human-caused conflicts, policy planning, emergency response and planning, natural hazards, public health, climate change, and humanitarian response.

## Supplementary information

Supplementary information

## Data Availability

The code used for this project was developed using Python and ESRI ArcMap (available at ESRI ArcGIS Desktop, https://www.esri.com/en-us/arcgis/products/arcgis-desktop/resources). A pseudo code is provided in the Methods section of this manuscript. Also, in the manuscript we have provided workflow diagrams across the years and pseudocode is provided in supplement information. Additionally, citations and 4 code snippets are available at 10.6084/m9.figshare.28439699. For additional details, including more comprehensive instructions, users can contact ORNL via email at landscan@ornl.gov. These resources will enable readers to replicate the LSG dataset.
