# Supplementary material for: LandScan Global 30 Arcsecond Annual Global Gridded Population Datasets from 2000 to 2022
Source: Sci Data. 2025 Mar 24;12:495. doi: 10.1038/s41597-025-04817-z (PMC11933676; doi:10.1038/s41597-025-04817-z)
Supplement: Supplementary file 1 — Supplementary information [file 41597_2025_4817_MOESM1_ESM.pdf]

Algorithm 1: Calculation of global gridded population at 30 arcsecond

Input: LS USA, LS HD
Output: LandScan Global dataset
1: Initialize empty database z
2: j ← number of countries
3: **for** $i = 1$ to j **do**
4: **if** ($country_i$ $has\ LS\ HD\ or\ LS\ USA$) **then**
5: **if** ($country_i$ $has\ LS\ HD$) **then**
6: l ← aggregate population in 3 arcsecond LS HD raster to 30 arcsecond raster
m ← simultaneously, calculate HD independent population (Figure 2)
n ← calculate percent change between l and m rasters
for $o = 1$ to cells in n do
if (manual verification failed for $cell_o$ in n) **then**
p ← replace $cell_o$ in l with $cell_o$from raster m
end if
end for
normalize p to CIAWFB
Append p to z

7: **else**
8: c ← aggregate nighttime population in 3 arcsecond LS USA raster to 30 arcsecond raster
9: d ← aggregate daytime population in 3 arcsecond LS USA raster to 30 arcsecond raster
10: $e = \frac{c+d}{2}$
normalize e to CIAWFB
Append e to z
11: **end if**
12: **else**
13: x ←Estimate population of $country_i$ based on steps in Figure 2
Append x to z
14: **end if**
15: **end for**
16: **return** z
